# Alternative Childhood Vaccination Schedules in Israel: A Mixed-Methods Study on Prevalence, Patterns, and Public Health Implications

**DOI:** 10.3390/vaccines14010067

**Published:** 2026-01-06

**Authors:** Efrat Sales, Eliya Cohen, Deena R. Zimmerman, Nadav Davidovitch, Alison McCallum, Keren Dopelt

**Affiliations:** 1 Department of Politics and Government, Ben Gurion University of the Negev, Beersheva 84105, Israel; 2 Conflict Management, Resolution and Negotiation Program, Interdisciplinary Studies Unit, Bar-Ilan University, Ramat Gan 52900, Israel; eliya7701@gmail.com; 3 Maternal Child and Adolescent Department, Israel Ministry of Health, Jerusalem 94467, Israel; deena.zimmerman@moh.gov.il; 4 School of Public Health, Faculty of Health Sciences, Ben Gurion University of the Negev, Beersheva 84105, Israel; 5 Azrieli Faculty of Medicine, Bar-Ilan University, 8 Henrietta Szold St, Safed 13115, Israel; nadav.davidovitch@biu.ac.il; 6 Centre for Population Health Sciences, Usher Institute, School of Population Health Sciences, College of Medicine and Veterinary Medicine, The University of Edinburgh, Edinburgh EH16 4UX, UK; alison.mccallum@ed.ac.uk; 7 Department of Public Health, Ashkelon Academic College, Ashkelon 78211, Israel; dopelt@bgu.ac.il

**Keywords:** vaccine hesitancy, alternative vaccination schedules, single-antigen vaccines, childhood immunization, public health policy, Israel

## Abstract

**Background/Objectives**: Vaccination programs are highly effective public health interventions, yet parental hesitancy toward combination vaccines has led to growing demand for alternative vaccination schedules, defined in this study as parental requests to split or replace recommended combination vaccines with single-antigen vaccines for non-clinical reasons. While parental attitudes have been widely studied, little empirical evidence exists on the real-world use of single-antigen vaccines and their public health implications in countries with otherwise high coverage. This study examined the prevalence patterns and parental motivations for requesting such alternative vaccination schedules in Israel, where national guidelines recommend specific combination vaccines, including measles-mumps-rubella-varicella (MMRV) and the pentavalent diphtheria-tetanus-pertussis–inactivated polio–Haemophilus influenzae type b (DTaP+IPV+Hib) vaccines, but informal accommodations exist. **Methods**: A mixed-methods design was employed: a retrospective cohort analysis of vaccination data from 2018 to 2021 (before and during the COVID-19 pandemic) focused on measles (first dose at 12 months) and pertussis (four-dose primary series), followed by semi-structured interviews with Maternal and Child Health clinic providers, policymakers, and parents. **Results**: Alternative vaccination schedules involving single-antigen measles or pertussis vaccines are occasionally used despite official policy, accounting for less than 1% of vaccinations overall. Outcomes include delayed administration, lower uptake of combination vaccines, and incomplete protection in certain groups. Parents cited safety concerns, fear of immune overload, and mistrust of authorities. These concerns were often amplified by misinformation, while providers described balancing parental preferences with the need for adequate coverage. **Conclusions**: This study provides new evidence on how vaccine hesitancy translates into service utilization, highlights the tension between individualized parental decision-making and contribution to collective health, and underscores the need for communication, policy strategies and service designs that sustain high coverage while addressing community-specific concerns.

## 1. Introduction

Modern vaccination programs against diseases that cause severe morbidity and mortality were introduced over 200 years ago [1]. Although improvements in living conditions and broader health systems have reduced exposure to vaccine-preventable and modifiable conditions and their case fatality rates, the expansion of vaccination programs alone has prevented an estimated 2–3 million deaths per year from diseases such as diphtheria, measles, and tetanus. Vaccination has also substantially reduced the burden of avoidable ill-health from conditions that can be prevented or mitigated through immunization [2,3,4,5,6]. All major multilateral organizations emphasize the critical importance of vaccinating children. Beyond being highly cost-effective compared with many other health interventions, the societal benefits of vaccination include its contribution to addressing antimicrobial resistance at the source, improving overall population health, reducing the risk of poverty, and mitigating the broader economic harms associated with infectious diseases. By preventing illness and reducing healthcare costs, vaccination reduces avoidable absence from education, work, and, for many people, helps maintain a steady income, thereby reducing poverty and sustaining economic stability at the household and national levels [7].

Immunization is a central component of primary medical care; however, organizing, delivering, and adhering to complex childhood immunization schedules can be challenging for both parents and healthcare providers, thereby increasing the burden of prevention and contributing to reduced vaccine completion rates. One aspect of reducing these barriers is the recommended use of combination vaccines (vaccines that include multiple antigens in a single injection), containing multiple antigens administered within a single formulation [8]. A systematic review found no significant differences in adverse events or safety outcomes between combination and single-antigen vaccines [9].

In contrast, alternative or “split” vaccine schedules are defined as parental requests to separate these antigens into smaller combinations via multiple injections or to use single-antigen vaccines instead of the recommended combinations [10] for non-clinical reasons. These schedules provide protection against only one or a limited selection of diseases. Their timing and administration differ from the standard schedule, particularly affecting combination vaccines such as measles, mumps, rubella, and varicella (MMRV) or diphtheria, tetanus, pertussis, inactivated polio, and Haemophilus influenzae type b (DTaP+IPV+HiB), with parents instead requesting monovalent or single-antigen formulations. The use of such alternative schedules represents a form of vaccine hesitancy, arising when parents believe that the routine immunization schedule, particularly combination vaccines, is neither safe nor effective [11]. This phenomenon is not new; however, earlier efforts to address it predated the global decline in vaccine uptake since 2019 and the spread of misinformation [12]. Vaccine hesitancy reflects broader issues of trust in health authorities, parental autonomy in medical decision-making, and the spread of misinformation in a “post-truth” era [13]. Situating alternative vaccine schedules within this framework allows us to examine medical and sociocultural dimensions of parental decision-making.

To conceptualize this phenomenon, the present study is informed by the World Health Organization Strategic Advisory Group of Experts (WHO SAGE) framework on vaccine hesitancy. This framework conceptualizes vaccine hesitancy as a context-specific and dynamic phenomenon shaped by multiple interacting dimensions, including confidence (trust in vaccines, health systems, and policymakers), convenience (accessibility and ease of vaccination services), and complacency (perceived risks of vaccine-preventable diseases) [14]. More recent extensions of the framework further emphasize the roles of calculation (active information seeking and risk assessment), and collective responsibility, reflecting the tension between individual decision-making and public health goals [15].

Previous studies have found that overall vaccination rates are higher with combination vaccines, such as the pentavalent vaccine (DTaP+IPV+HiB) or the MMR vaccine, 86% compared with 50% for single-antigen vaccines, such as IPV, measles, or DTaP in the United States [16]. In China, one study reported that completion of the three-dose Haemophilus influenzae type b (HiB) vaccine was three times higher among children receiving combination vaccines (pentavalent) compared with those receiving HiB alone [17]. Research examining parents’ perceptions, attitudes, and beliefs about combination versus alternative vaccination schedules indicates persistent concerns that combination vaccines, because of their multiple antigens, may overload the immune system and harm the child’s health [18]. Other concerns include fears that they reduce the immune system’s ability to respond effectively to disease, general safety doubts, and mistrust of physicians and the broader health system [19,20].

Parental perceptions, attitudes, and beliefs regarding vaccines, including combination vaccines, are often shaped by misinformation and limited knowledge, frequently derived from websites or social media platforms. In the United Kingdom, disinformation about the safety of the MMR vaccine and concerns over adverse events were among the main reasons cited for adopting alternative vaccination schedules [21,22]. The content of misinformation also varies by vaccine type; for example, parental concerns about immune overload have been reported more frequently with the DTaP+IPV+HiB vaccine [23]. Furthermore, the COVID-19 pandemic and the introduction of COVID-19 vaccines amplified the spread of misperceptions and disinformation about the safety and efficacy of routine childhood immunizations, thereby increasing parental hesitancy [24]. In addition, parental mistrust has been reinforced by broader perceptions that physicians and health authorities may not always communicate transparently or may be influenced by external interests, further contributing to vaccine hesitancy [25]. While concerns regarding vaccine safety, misinformation, and mistrust in health authorities have been documented in the literature for over a decade, the COVID-19 pandemic appears to have marked a qualitative shift in their scope, visibility, and societal impact. Recent evidence from the COVID-19 pandemic further demonstrates that public health work is no longer limited to providing biomedical information. It requires advanced competencies in managing public trust, addressing misinformation, and making ethical decisions in uncertain conditions [26].

The prevalence and emphasis of parental concerns vary over time, often influenced by media coverage or specific events that trigger doubt, across different communities, where skepticism may be tied to religious or cultural factors, and across regions and countries, particularly in contexts where mistrust of government or doubts about health system functioning are widespread [13,25]. Consequently, some parents may seek out providers who offer alternative vaccine schedules that rely on single-antigen vaccines rather than the recommended combinations [27]. The use of single antigen, alternative combinations, and reduced dosage for non-clinical reasons is rarely an official policy but is accommodated by individual clinicians (often privately) in some countries. This may occur in an attempt to address vaccine hesitancy and thus reduce the proportion of unvaccinated children lost to services [25].

The Israeli case is particularly important: the country has historically high vaccination coverage and a well-developed public infrastructure of maternal and child health centers, known as Tipat Halav (“Drop of Milk,” from the original French Gout de Lait). Routine childhood vaccinations in Israel are administered primarily in Tipat Halav clinics, a national network of preventive healthcare centers for infants and young children. The formal policy of the Ministry of Health is to vaccinate children with combination vaccines such as MMRV and DTaP+IPV+HiB [28]. However, in practice, informal accommodation allows parents to request single-antigen or split schedules for non-clinical reasons. Despite extensive international evidence on the safety and effectiveness of combination vaccines, limited empirical research has examined the real-world implementation of such alternative schedules within Israel’s immunization system. This gap is especially relevant in a country with otherwise high coverage, where even small shifts in parental behavior may undermine herd immunity and increase the risk of outbreaks. Although national vaccination rates are high, gaps often cluster within specific communities; therefore, localized declines or delays in completion of vaccination can create pockets of under-immunized children, limit community protection and enable rapid spread of infection Even a relatively small group of under-vaccinated children can generate an avoidable burden of ill-health in families and communities, disproportionate societal and economic burden, including increased parental work absences, productivity losses, hospitalizations, and substantial healthcare expenditures [29,30].

This study aims to examine the phenomenon of alternative vaccination schedules by combining quantitative and qualitative methods to assess the prevalence patterns, and reasons parents request to split combination vaccines. We investigated the use of alternative vaccine schedules and the relationship with the reduction in vaccine doses administered in Israel between 2018 and 2021, before and during the COVID-19 pandemic. In light of recent outbreaks of pertussis and measles in Israel, and consistent with WHO data indicating increased outbreaks of measles, diphtheria, mumps, and pertussis in Canada, the United States, and the EU in 2024 compared with the previous five years (2018–2023) [6], we focused specifically on the first dose of the measles-containing vaccine and the primary series of pertussis. The measles vaccine is included in the combination vaccine MMRV, administered at 12 months. Pertussis is part of the Pentavalent vaccine (Diphtheria, Pertussis, Tetanus, Hepatitis B, and Hib), with four doses administered at 2, 4, 6, and 12 months. Accordingly, our research questions were as follows: (1) What is the prevalence of requests for alternative or split vaccine schedules in Israel? (2) Which demographic, cultural, and socioeconomic factors are associated with such requests? and (3) How do these practices affect timely and complete immunization? By examining the scope and characteristics of these practices, this study provides new insights into how vaccine hesitancy translates into service utilization, with direct implications for public health policy and strategies to improve vaccine uptake.

## 2. Materials and Methods

### 2.1. Study Design

We employed an explanatory sequential mixed-methods design, in which quantitative findings informed the subsequent qualitative inquiry [31]. The quantitative component was the primary source of evidence, and the qualitative component provided complementary and additional insights. The design followed an explanatory sequential logic: quantitative results informed the qualitative inquiry.

### 2.2. Quantitative Component

#### 2.2.1. Data Sources and Population

In the first stage, a retrospective cohort study was conducted using routine data on children who received pertussis and measles vaccinations in maternal and child health centers (“Tipat Halav”) in Israel between 2018 and 2021. The study included all children born between 2018 and 2021 with at least one visit to centers operated by the Ministry of Health, the municipalities of Tel Aviv and Jerusalem, and Leumit Health Services, which together cover approximately 70% of the registered child population [32]. Inclusion criteria were children who attended at least one Maternal and Child Health clinic visit during the study period and had available vaccination records for measles and/or pertussis. Children who did not attend any Maternal and Child Health clinics, who received care exclusively through other healthcare providers, or whose vaccination records were incomplete or missing were excluded from the analysis. Data were extracted from the electronic medical records stored within the Ministry of Health’s Machshava Briah (Healthy Thinking) database. All data were anonymized prior to researcher access.

#### 2.2.2. Variables and Measures

Children were classified by target vaccination year (including those up to 12 months plus 5 weeks) rather than by birth year [3]. Vaccination status was categorized as on time, late (more than 5 weeks after the target age), or not vaccinated. We distinguished between receipt of single-antigen measles vaccine versus MMRV, and between single-antigen pertussis vaccine versus combination vaccines [33]. To assess the robustness of this operational definition, sensitivity analyses were conducted using alternative delay thresholds (≥4 weeks and ≥8 weeks) and by modeling delay as a continuous variable, defined as the number of weeks elapsed from the target vaccination age.

Demographic covariates included socioeconomic status (SES), religious/ethnic affiliation, and region (as measured by the central-periphery index). SES was determined according to the socioeconomic classification of the locality in which the Maternal and Child Health Center is located. Religious/ethnic affiliation (general Jewish, Arab, ultra-Orthodox, others) was obtained from the Ministry of Health’s internal registry linked to each child’s medical record. The other population includes people not categorized as either Jewish or Arab. Most are people who made Aliyah under the Law of Return, so are socially and politically integrated into the Jewish rather than the Arab population, although they are not halachically Jewish according to the state’s orthodox interpretation of Jewish law. In CBS tables on religious identity, they are typically coded as “lacking religious affiliation.” This category also includes people without formal national or civil status, such as labor migrants, asylum seekers, and individuals with unclear or unresolved legal status. This variable reflects an official administrative classification and is not based on parental declaration.

The central-periphery index is an official Israeli geographic classification that reflects a locality’s distance from major metropolitan centers and its access to essential services, functioning similarly to international urban–rural classifications. Both the SES and central–periphery indices were assigned according to Central Bureau of Statistics [32] classifications for each locality, based on the geographic location of the Maternal and Child Health Centers, using data extracted from CBS records for 2018–2021.

To assess the impact of the initial phase of the COVID-19 pandemic (before completion of the first round of vaccination), we compared 2018–2019 with 2020–2021. We restricted the quantitative analysis to 2018–2021 because complete, validated national vaccination data for 2022–2023 were not yet available at the time of analysis. These years also capture the initial phase of the COVID-19 pandemic, during which delays in routine vaccinations were documented by clinic staff.

#### 2.2.3. Quantitative Analysis

Descriptive analyses, frequency distributions, and chi-square tests were conducted to assess prevalence and variables associated with split and alternative vaccination schedules using SPSS v29 (IBM, Armonk, NY, USA). Sensitivity analyses were performed to examine whether the observed associations between split vaccination schedules and vaccination delay were robust to alternative specifications of delay. Results were compared across categorical and continuous operationalizations of delay. Because this analysis was based on all children recorded in the Ministry of Health’s national administrative databases, representing the full population of children who utilized public well-child services during the study period, confidence intervals were not required. Although this population reflects approximately 70% of all children in Israel, it constitutes the entire population of service users rather than a sampled dataset; therefore, *p*-values were used to describe group differences, while recognizing that statistical significance in large administrative datasets does not necessarily imply substantive or public health relevance.

### 2.3. Qualitative Component

#### 2.3.1. Interview Design and Development

In addition to the cohort study, a qualitative component was incorporated to provide contextual and interpretive insights into the phenomenon of alternative vaccination schedules. This qualitative element was not designed as a standalone investigation but rather as a complementary source of evidence to enrich the quantitative findings and to explore underlying organizational, professional, and parental perspectives. The qualitative component was conceptually informed by the WHO SAGE framework [14] on vaccine hesitancy, which guided the development of the interview domains and the interpretation of findings, with particular attention to issues of trust, information exposure, risk appraisal, and parental autonomy.

Two separate semi-structured interview guides were used in the qualitative component of the study. While the overall design was explanatory sequential, the parent interviews intentionally included an inductive element to avoid framing effects and to allow for issues related to split vaccination to emerge without direct prompting, thereby complementing the explanatory aims of the mixed-methods framework.

(1) The guide for Maternal and Child Health nurses and policymakers included 15 questions, of which two explicitly addressed routine split vaccination schedules.

(2) The guide for parents included 20 questions and did not contain any direct items regarding split vaccination; however, the topic emerged spontaneously in several interviews, highlighting its perceived relevance among participants. The use of approximately 20 core questions ensured comprehensive coverage of key conceptual domains while remaining feasible within the planned interview duration. Semi-structured probes were applied flexibly, allowing interviewers to adapt the depth and sequencing of questions based on participant role and responses.

Both interview guides were developed by the research team and underwent content validation by two public health physicians and two public health nurses. The validation process focused on assessing the relevance, clarity, and comprehensiveness of the interview questions in relation to the study objectives. Reviewers were asked to evaluate whether the questions adequately captured key dimensions of childhood vaccination practices, parental decision-making, and policy considerations, and to suggest modifications where needed. Based on their feedback, minor revisions were made to the wording, sequencing, and scope of several questions to improve clarity and ensure content validity prior to data collection.

Individual semi-structured interviews were selected over focus groups to enable participants to discuss potentially sensitive vaccination decisions in a private setting, to minimize social desirability and conformity pressures, and to accommodate the heterogeneous roles of parents, nurses, and policymakers.

In this study, ‘public health professionals’ referred to Maternal and Child Health (Tipat Halav) nurses, while ‘policymakers’ referred to district- and national-level Ministry of Health officials responsible for childhood vaccination policy. Interviews were conducted by ES and EC, both doctoral students in the Department of Politics and Government and in the Conflict Management, Resolution, and Negotiation Program. All interviews were conducted in Hebrew. All participants were fluent Hebrew speakers; therefore, no interpreters were required, and no participants were excluded based on language proficiency.

#### 2.3.2. Participants

The qualitative component included interviews with both professionals involved in childhood vaccination policy and delivery, as well as parents.

Professionals: Participants were recruited using purposive sampling based on their roles and experience in childhood immunization policy or service delivery. This purposive sampling strategy ensured representation of perspectives from both frontline service provision and policy-making levels. The nurse sample included Maternal and Child Health nurses employed by the Ministry of Health, as well as by Tel Aviv and Jerusalem municipalities, and by Health Maintenance Organizations (HMOs). Participants were drawn from all geographic districts in Israel. The professional sample comprised 20 Maternal and Child Health nurses (including Jewish, Druze, Bedouin, and Arab nurses) and 10 policymakers at the district and national levels of the Ministry of Health.

Parents: A total of 20 parents participated in the study. Parents were recruited through social media advertisements and snowball sampling. The sample included Jewish parents from both the general (secular/traditional) population and the ultra-Orthodox sector. Participants were drawn from diverse geographic regions across Israel and represented a range of socioeconomic backgrounds, allowing for variation in lived experiences and perspectives related to childhood vaccination decisions. Despite targeted efforts to recruit vaccine-hesitant Arab parents, none were identified. This aligns with the findings of this study and Ministry of Health reports, which document high vaccination coverage and low levels of vaccine hesitancy among Arab parents in Israel compared with the Jewish population. Inclusion criteria were having at least one child eligible for routine childhood vaccination.

Interviews continued until thematic saturation was reached, defined as the point at which no substantively new themes related to vaccination decision-making or perceptions of alternative vaccination schedules emerged in successive interviews.

#### 2.3.3. Qualitative Analysis

All interviews were audio-recorded, transcribed verbatim, and thematically analyzed following Braun and Clarke’s [34] approach. Analysis proceeded iteratively alongside data collection. Initial coding was conducted after early interviews, and emerging codes and themes were reviewed continuously. Recruitment continued until no substantively new codes or themes were identified, with additional interviews conducted to confirm data saturation.

Thematic analysis followed the six phases outlined by Braun and Clarke [34]: familiarization with the data through repeated reading of transcripts; generation of initial codes; identification of candidate themes; review and refinement of themes; definition and naming of final themes; and reporting with representative quotations. Coding was primarily inductive while informed by the conceptual framework. Data were analyzed using ATLAS.ti (version 25; Berlin, Germany). Because the Hebrew-language version of the software does not include AI-assisted analytic functions, all coding, categorization, and theme development were performed manually. Coding was conducted independently by two researchers, with discrepancies resolved through discussion and consensus. In line with the recommendations of ENTREQ (Enhancing Transparency in Reporting the Synthesis of Qualitative Research), we report the rationale for including qualitative data, the interview process, participant selection, and the analytic approach [35].

### 2.4. Integration of Methods

While the qualitative findings were not intended to generate standalone conclusions, they played an important role in contextualizing and clarifying the cohort results by illuminating parental hesitancy, organizational constraints, and policy considerations surrounding fragmented vaccination practices. Integrating quantitative and qualitative evidence strengthens the robustness of the study and provides a more comprehensive understanding of both the prevalence and the drivers of the phenomenon.

### 2.5. Ethical Considerations

The data analysis was part of a larger study on childhood vaccination, for which approval was granted to ND, ES, and EC. Ethical approval for the quantitative component of the study was granted by the National (Supreme) Helsinki Committee of the Israel Ministry of Health, which provided fully anonymized and de-identified datasets for analysis (Approval No. COR-MOH-089-2021; Date: 11 August 2021). The qualitative interviews with Maternal and Child Health nurses and national policymakers were approved by the Ethics Committee of Ben-Gurion University of the Negev (Approval No. 2024/19; Date: 4 December 2024). The qualitative interviews with parents were approved separately by the Ethics Committee of Bar-Ilan University (Approval No. ISU202402003; Date: 14 April 2024). No amendments were required for any of the approved protocols.

The research team did not have access to any identifiable or raw individual-level data. All datasets provided by the Ministry of Health were fully anonymized prior to transfer, in accordance with GDPR-equivalent standards and the Five Safes principles (Regulation (EU) 2016/679). To ensure that no data could be linked or traced back to individual children, records were assigned unique identification numbers, and multiple layers of separation were maintained between the ID keys and the corresponding content (“payload”) data. This multi-step process ensured effective anonymization and prevented any possibility of re-identification. All Ministry of Health datasets were stored on a password-protected, restricted-access secure server, in accordance with national data protection procedures.

For the qualitative component, all participants received written information about the study and provided signed informed consent prior to participation, explicitly agreeing to audio-recording and verbatim transcription. All interview data were anonymized immediately following transcription. As is standard in semi-structured interviews, the interviewers (ES and EC) necessarily knew the identity of participants during data collection; however, all transcripts were fully de-identified prior to analysis, and the code list linking identifiers to transcripts was stored separately to prevent any possibility of re-identification. All study procedures complied fully with the approved ethics protocols.

## 3. Results

### 3.1. Quantitative Study

#### 3.1.1. Demographics

Between 2018 and 2021, a total of 728,733 children were born in Israel. Of these, 499,975 (68.6%) who attended at least one visit to child healthcare centers operated by the Ministry of Health, the municipalities of Tel Aviv and Jerusalem, and Leumit Health Fund were included in the study sample. In total, the study covered 498 healthcare centers registered in the Ministry of Health’s “Machshava Bri’ah” electronic health records system.

A χ^2^ test for goodness of fit was used to compare the study population with the general child population in Israel across demographic variables: religion, birth year, socioeconomic status (SES), and peripherality index (based on the child’s place of residence and healthcare center). All variables were statistically significant (*p* < 0.001).

Table 1 presents a comparison of demographic characteristics between the study population and the general child population.

In Israel, all Maternal and child health clinics (“Tipat Halav”) services operate exclusively within the public healthcare system, with no private well-child clinics. The study population included all children who attended at least one visit to a public well-child clinic during the study period, representing approximately 70% of the national child population. Therefore, the denominator for the study reflects children actively engaged with preventive child health services, rather than the entire child population in Israel.

#### 3.1.2. Prevalence and Timing of Alternative Vaccination Schedules

In total, 499,975 children born between 2018 and 2021 visited at least one of the 498 maternal and child health centers. Between 2018 and 2021, 4319 (0.9%) children received any type of split vaccine.

**Figure 1:**
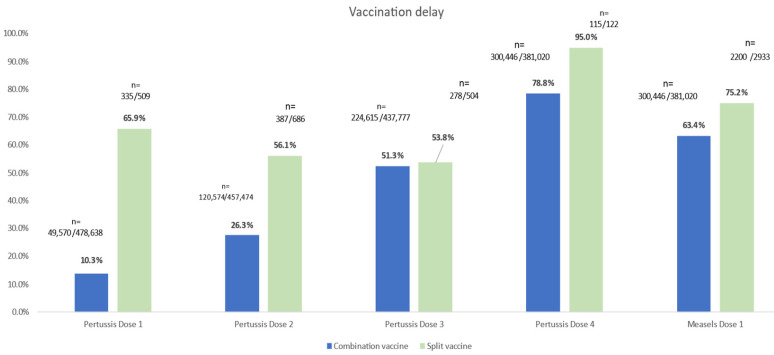
Vaccination Delays by Vaccine Type (Combination vs. Split) among children vaccinated for pertussis (n = 686) and Measles (*n* = 2933).

Figure 1 illustrates the difference in vaccination delays between children who received combination vaccines and those who received split vaccines for pertussis and measles. Across all doses, delays were substantially more common among children vaccinated with split vaccines. For example, 65.9% of children receiving the first dose of the pertussis split vaccine experienced a delay, compared with only 13.9% among those who received the combination vaccine. A similar pattern was observed for measles (75.2% vs. 63.4%). These findings indicate that split vaccination schedules are associated with greater delays in completing the recommended immunization series.

Sensitivity analyses demonstrated that the association between split vaccination schedules and delayed vaccination persisted across alternative operationalizations of delay. Using both stricter (≥4 weeks) and more lenient (≥8 weeks) cut-offs, as well as modeling delay as a continuous variable (in weeks from the target age), children receiving single-antigen vaccines consistently exhibited longer delays compared with those receiving combination vaccines. The direction and magnitude of the associations remained stable across specifications.

#### 3.1.3. Pertussis

Of those immunized, 686 children (0.14%) were given the pertussis single-antigen vaccine and were not immunized with the standard pentavalent vaccine (DTaP+IPV+HiB) (Table 2). The proportion of children receiving pertussis-only vaccination declined from the first to the fourth dose, from 0.15% for the second dose to 0.03% for the fourth. This was because most children who did not receive the standard combination vaccine did not complete the schedule. In addition, most of these children were vaccinated at least five weeks later than the target date. In contrast, significant delays were observed only for the third and fourth doses among those who received the pentavalent vaccine.

We also found differences in the proportions of children receiving the pertussis-only vaccine between religious/ethnic groups, SES (Table 2 and Table 3). More of those who received the pertussis split vaccine were in the low and medium SES groups compared to the high SES group, with the difference being greatest for the third and fourth doses. Receipt of pertussis-only vaccine was 44% for children from low and medium SES groups versus 11.9% for those from high SES (*p* = 0.017).

The use of pertussis-only vaccines also varied according to the religious/ethnic community. The general Jewish and Jewish ultra-orthodox populations comprised a greater proportion of those receiving the pertussis-only vaccine (all doses) compared to Arab and other communities. For the second dose, 62.3% were classified as members of the general Israeli Jewish population, 17.7% were Jewish ultra-orthodox, and 6.7% were classified as Arab (*p* < 0.001). Pertussis-only vaccination was also highest in those geographical areas classified as peripheral. Here, the proportion increased with each dose received from 44.1% for dose 1 to 60% for dose 4. However, it was lowest in the far periphery at 10.6% for dose 4. In addition, use of the pertussis-only vaccine was greater for children that belonged to the 2018–19 pre-pandemic cohort. Receipt of pertussis-only vaccine for the second dose of pertussis was 63.8% in 2018–2019 compared to 36.2% in 2020–2021 (*p* = 0.002).

#### 3.1.4. Measles

Of 499,975 children, 2933 (0.59%) received a Measles split vaccine rather than the routinely used MMRV vaccine in Israel. Vaccination more than 5 weeks after the target date occurred in 75.1% of children who received the Measles split vaccine, compared with 63.4% among those who received the MMRV vaccine.

Similarly to the pertussis-only vaccine, we found a difference between religious/ethnic groups, socio-economic status, and geographical area of children receiving the measles split vaccine (Table 2 and Table 3). Of those receiving the measles split vaccine, 45.4%, were in the low-medium SES groups compared to 9.1% classified as high SES (*p* > 0.05). The variation by religious/ethnic group followed a similar pattern to pertussis, with receipt of split vaccination being 63.6% in the general Jewish population, 25.9% in the Jewish ultra-orthodox communities, and 6% in the Arab and other communities (*p* = 0.003). Variation by geographical area was again highest in the peripheral areas (47.1%), in the center areas (42.8%), with lower use in the far peripheral areas at 10.1% (*p* = 0.05). Similarly to pertussis, the proportion of children receiving split measles vaccine (first dose) was greater for children who belonged to the 2018–19 pre-pandemic cohort and was 75.2% in 2018–2019 compared to 24.8% in 2020–2021 (*p* < 0.001).

### 3.2. Qualitative Study

Public health nurses working in maternal and child health centers, policymakers, and parents described their experiences and perspectives regarding requests for split or alternative childhood vaccination schedules in Israel. Across interviews, participants referred to variations in such requests across population sectors and to the considerations that shape parental decisions and professional responses.

The qualitative analysis identified three main themes: (1) the prevalence of split vaccination across population sectors; (2) reasons for requesting split or alternative vaccination schedules; and (3) participants’ views on policy flexibility regarding childhood vaccination.

#### 3.2.1. Prevalence Across Population Sectors

Participants described variation in requests for split or alternative vaccination schedules across population sectors. Nurses reported that such requests were more commonly raised by Jewish parents, particularly from ultra-Orthodox and secular communities. In many cases, nurses noted that they were able to encourage parents, most often mothers, to ultimately adhere to the standard combination vaccination schedule.

“There are quite a few from the ultra-Orthodox community, and I’m usually able to convince them to vaccinate.”

(Nurse, Maternal and Child Health Clinics in the Jerusalem district)

Some parents perceived the interaction surrounding vaccination decisions as pressuring or guilt-inducing. These experiences may reflect communication challenges and systemic constraints in busy clinic settings, rather than intentional coercion.

“…Then there was an argument with her, diphtheria or not, about whether it’s possible to separate all the vaccines and come each time individually. And then she tells me, ‘I’m opening a special vaccine dose just for you […]. In short, she put me under a lot of stress and made me feel guilty and remorseful, which I am quite sensitive to […] It was terrible, truly terrible. I remember it as a very unpleasant experience.”

(Interviewee 1, Northern District)

“There was also the tetanus vaccine. They refused to give it to me separately and started causing problems, so in the end I took the tetanus together with the pentavalent vaccine.”

(Interviewee 2, Haifa district)

In contrast, such requests were rare among Israeli Arab and Druze parents, who generally demonstrated higher levels of trust in the healthcare system.

“No, not really, there’s no split. There’s trust in the system and in us, and a lot depends on how you present it.”

(Nurse, Maternal and Child Health Clinics in the North district)

#### 3.2.2. Reasons for Vaccine Splitting

Participants identified four main reasons why some parents request split or alternative vaccination schedules: (1) misinformation and social media influence; (2) concerns about vaccine safety; (3) declining trust in the Ministry of Health, especially after the initial phase of the COVID-19 pandemic and immunization schedule; and (4) recommendations from private or alternative practitioners who recommend individualized vaccination plans.

(A) Misinformation and Social Media Influence—Many nurses and policymakers highlighted the strong influence of misinformation circulating on social media platforms in shaping parental attitudes toward childhood vaccination. Parents were often described as forming opinions based on viral content shared by bloggers, online groups, or influencers rather than on professional medical guidance.

“No, it was quite broad, including people from different sectors. Some were more educated, while others seemed less informed—they had seen a video on TikTok or Instagram, where a blogger talked about her son or brother, and suddenly she became a kind of ‘vaccine expert,’ and people just followed along.”

(Nurse, Maternal and Child Health Clinics in the Central district)

“All this idea of split vaccines isn’t really based on facts; it mostly stems from rumors and social media. Many online groups share and amplify misinformation, and suddenly one person’s comment spreads like wildfire, turning into false and unfounded claims.”

(Policymaker)

Similarly, in the interviews with the parents themselves, they reported that their sources of information regarding routine vaccinations were mainly various websites and social media platforms that were not affiliated with official health authorities.

“Yes, I’m in a Facebook group […] it’s not an anti-vaccine group, but they do talk about all the vaccines, both for and against. Each vaccine is discussed separately, weighing its benefits against its possible harms.”

(Interviewee 4, Southern District)

“There was a website, though I no longer recall its name, from which we read information about the vaccines. Based on what we saw there, and on our intuition, we felt even a slight possibility of neurological or cognitive harm to our child was enough for us to decide not to vaccinate.”

(Interviewee 1, Northern District)

(B) Concerns about the safety of vaccines—Policymakers and nurses reported that some parents expressed concern about vaccine safety as a common reason for requesting split vaccination schedules. They noted parental fears related to “overloading” their children’s immune system or fear of possible side effects, such as autism or allergic reactions.

“She told me, ‘I don’t want to overwhelm his immune system, I prefer that he gets a little at a time.”

(Nurse, Maternal and Child Health Clinics in the Tel Aviv district)

These concerns were also raised in interviews with the parents themselves, which often led parents to ask that the vaccines be given separately.

“When I vaccinated them for measles, I asked for measles only, not the combined shot. I’m very worried about this vaccine […] about the possible neurological damage it can cause, the harm to the body. Some children became disabled afterward, children with autism, I’ve heard quite a few stories.”

(Interviewee 3, Haifa District)

(C) Declining Trust in Health Authorities—Many nurses, policymakers, and the parents themselves described how trust in the Ministry of Health declined during and after the acute phase of the COVID-19 pandemic, as parents questioned frequent changes in vaccine guidelines and information provided by the authorities.

“For instance, there’s a mother who’s a teacher. She vaccinated her older children with the combined vaccines, but following the COVID-19 pandemic, with her youngest child, she said she’d lost trust in the system, and in us, and now prefers to separate the combination vaccine.”

(Nurse, Maternal and Child Health Clinics in the Southern district)

“You already understand from the beginning that if there’s some kind of pressure here, that you’re being pressured, there’s probably something very, very bad here, something very, very stinky.”

(Interviewee 5, Southern District)

“Unfortunately, with the COVID-19 vaccine, I felt that there had not been enough time to examine it thoroughly. That made me even more alert and cautious, and less willing to accept information uncritically. It pushed me to keep reading and looking for more information. […] I don’t have much trust anymore.”

(Interviewee 5, Central District)

(D) Recommendations from Private or Alternative Practitioners—Finally, several nurses and policymakers noted that some parents follow the advice of private physicians or alternative health practitioners who recommend personalized vaccination plans that deviate from national guidelines.

“Some parents, despite all explanations, still prefer to split the vaccines. In certain cases, they consult a physician who provides an alternative vaccination schedule and advises them to separate the combined vaccines.”

(Nurse, Maternal and Child Health Clinics in the Tel Aviv district)

The parents themselves also noted that they followed the recommendation of an alternative healthcare practitioner to administer the vaccines in a split schedule.

“We also consulted with an alternative healthcare practitioner who had studied the topic of vaccines, and together we developed a personalized vaccination plan tailored to our family background. As a result, our child received the vaccines in a fragmented and significantly delayed manner, and some vaccines were not administered at all.”

(Interviewee 6, Haifa District)

“But he said in parentheses […] it may be that you only need four vaccines in the pentavalent, but you can’t get them like that in a Maternal and Child Health Clinics.”

(Interviewee 7, Jerusalem district)

“We consulted a physician in an alternative clinic, who is also a conventional doctor and integrates both approaches, and he developed a vaccination plan for us. He explained which vaccines he considered essential, which he recommended skipping, and which could be postponed. Based on his guidance, we decided to split and delay the vaccines.”

(Interviewee 2, Haifa District)

#### 3.2.3. The Attitudes of the Policymakers and Nurses Toward the Alternative Childhood Vaccination Schedules Policy

Except for one participant, all interviewees expressed agreement with the Israeli Ministry of Health’s policy that allows for flexibility when parents insist on split vaccination. They emphasized that, although combined vaccines represent the optimal schedule, it is preferable to permit parents to separate doses rather than risk complete refusal of the vaccine. According to the participants, this approach ensures that children remain at least partially protected against some of the diseases, reflecting a pragmatic balance between public health priorities and parental concerns.

“Look, it’s not just about what I think; we had to make those decisions in the midst of a polio outbreak. For example, we encountered parents who refused the combined pentavalent vaccine, so we allowed them to split it and give only the IPV vaccine. For me, it was important that the child be at least partially protected. I know it’s not the ideal solution, but it’s better than not vaccinating at all.”

(Policymaker)

“We don’t argue with parents; it’s their child, after all. If they insist, we agree to split the vaccine. What matters most is that the child is protected at least against some of the diseases.”

(Nurse, Maternal and Child Health Clinics in the Jerusalem district)

This theme also emerged in the interviews with parents, who emphasized the importance of maintaining the parents’ right to freely choose which vaccines to administer to their children and when.

“We don’t see it in black and white, but we want to think and make an independent decision, that no one dictates to us. We want to decide which vaccine and when. And if we prefer to do it gradually, not to give everything at such a young age, but to spread it out over more years.”

(Interviewee 1, Northern District)

Overall, the qualitative findings highlight that requests to split combined childhood vaccines were described across diverse population groups in Israel. Participants referred to a range of interconnected considerations shaping parental decisions, including sources of information, safety concerns, trust in health authorities, and professional advice. Across interviews, vaccine splitting emerged as one expression of parental hesitancy toward routine childhood immunization.

## 4. Discussion

In this study, we examined the phenomenon of alternative vaccination schedules by considering findings from two cohorts drawn from routine vaccination data and semi-structured interviews with parents, health professionals in the clinics, and policymakers. Our findings show that requests for split vaccination remain relatively uncommon overall but are concentrated within specific population sub-groups. The variations in split-vaccine requests and vaccination delays across SES groups, regions, and sectors likely stem from differences in trust, cultural norms, misinformation exposure, and health-seeking behaviors. These requests appear to be driven by reported safety concerns, broader distrust in authorities, and parents’ desire to assert autonomy during clinical encounters, particularly in contrast to situations in which their autonomy might be restricted due to isolation or quarantine requirements following exposure. If unaddressed, this situation risks creating a vicious cycle of pockets of under-protection, avoidable outbreaks, harm, death, and further mistrust in authorities. The local and international resurgence of measles and pertussis outbreaks since 2024 underscores the urgency of addressing factors that contribute to under-vaccination and declining population immunity.

Interpreted through the WHO SAGE vaccine hesitancy framework, our findings suggest that requests for alternative vaccination schedules reflect the interaction of reduced confidence in health authorities, heightened calculation driven by misinformation, and tensions between parental autonomy and collective responsibility [14,15]. This interpretation is consistent with previous research, which shows that vaccination decisions are shaped not only by clinical considerations but also by broader social and institutional factors, including trust in health authorities, perceived risk, and norms of collective responsibility [36].

Previous studies on non-clinical use of alternative schedules largely predate the widespread influence of social media and the recent global decline in routine immunization coverage. Revisiting the upsurge in requests for alternative vaccine schedules and split vaccines in the current context is therefore essential. Our analysis revealed that alternative schedules are associated with delayed administration and reduced completion rates [37,38]. Importantly, their use is not randomly distributed across the population but clustered within specific social, ethnic, and religious groups. Similar patterns were reported in the UK, where Pearce et al. [22] found that requests for separated or delayed vaccines contributed to reduced coverage and growing inequities. This clustering creates concentrated pockets of unvaccinated and under-vaccinated children, often overlapping with geographical inequities. Such uneven coverage increases vulnerability, elevates the risk of localized outbreaks, and undermines community protection, particularly for individuals who cannot be vaccinated for clinical reasons, fail to mount an immune response, or are too young for immunization. These findings align with earlier studies that document lower acceptance and greater delays for children receiving single-antigen vaccines compared with those following combination schedules [16,22], reinforcing concerns about the public health risks associated with non-standard immunization practices.

It is important to recognize that the choice of alternative vaccine schedules may not stem solely from concerns about child health. It may also reflect a broader trend of “intensive parenting,” in which parents assume responsibility for making complex medical decisions independently rather than discussing concerns and developing a shared understanding with a trusted professional. The perception that parents are on their own can undermine confidence in public health authorities and normalize individualized or alternative vaccine schedules as a form of empowered and responsible parenting [39,40]. The findings from our semi-structured interviews reinforce this perspective: parental decision-making was shaped not only by safety concerns but also by perceived autonomy, distrust in institutions, and the influence of community norms [19,41]. In Israel, vaccine behavior reflects long-standing negotiations between communities and the state, in which religious and ethnic identities play a central role [42].

In this context, Maternal and Child Health Clinics in Israel play a central role in mediating between parental concerns and national vaccination policy. As documented in previous studies, including in the UK, the provision of alternative schedules in response to parental requests has sometimes been used as a strategy to maintain engagement and reduce the risk of children remaining completely unimmunized [22,41,43]. However, evidence from our study indicates that alternative vaccination schedules, specifically the use of single-antigen measles and pertussis-only vaccines, do not reliably improve uptake.

Furthermore, our findings show that in Israel, many children who began an alternative vaccination course did not complete the recommended series, particularly for pertussis, highlighting a pronounced risk of under-vaccination. Part of this dynamic relates to the role of alternative advisers in shaping parental decisions. In Israel, alternative or private practitioners are not authorized to administer vaccines; however, they can and do provide parents with individualized vaccination plans that deviate from the Ministry of Health’s recommendations. Parents then bring these plans to Maternal and Child Health clinics to request delayed or split administration. This dynamic stands in contrast to the United Kingdom, particularly Scotland, where only licensed providers may administer or advise on immunization schedules under the National Health Service (Scotland) Act 1978, and private practitioners cannot recommend non-standard vaccination pathways for routine childhood vaccination [44]. When private providers offer alternatives that deviate from national recommendations, it reinforces the perception that the standard schedule is only one of several equally valid options. This weakens the messaging of public health professionals and may incentivize parents to prioritize individualized care over collective health protection, posing a significant challenge for equity in immunization programs [45].

These concerns also reflect deeper gaps in access to reliable scientific information, limited parental involvement in health decision-making processes, and structural features of vaccination programs [18,23]. Addressing these gaps requires tailored strategies across contexts. In Israel, trusted messengers within religious and ethnic communities may play a particularly effective role [46,47]. Our interviews revealed that in religious communities, decisions are often mediated through communal leaders such as rabbis [38]. In Israel, religious leadership is centralized and highly influential, which may further shape how vaccine information is mediated within these communities. Research in medical ethics suggests that, in certain communities, perceived control over a child’s body is deeply tied to parental identity and that public health messaging must consider these symbolic dimensions when engaging with vaccine-hesitant parents [48].

Responding to requests for single-antigen vaccines or alternative schedules for non-clinical reasons raises important ethical and policy challenges. The long-term impact of disinformation on trust in vaccination, uptake, and coverage rates is clear [49]. The ease with which outbreaks occur in under-vaccinated communities highlights the importance of conveying the shared responsibility to prevent the spread and risk of serious vaccine-preventable and modifiable illness, particularly the requirement to protect people unable to benefit from vaccination [50], including those in their own communities. Designing immunization programs that optimize uptake, coverage, and acceptance is complex and requires careful consideration, balancing individual and collective rights and public health ethics, including the role of vaccination in securing the right to health, education and family life. Importantly, the findings highlight how informal accommodations, such as allowing for parental choice for non-standard schedules, may unintentionally legitimize or institutionalize alternative practice. In Israel, where informal requests for alternative vaccine schedules are often honored, this may blur the boundary between official policy and practice.

The use of alternative vaccination schedules is a global phenomenon, but the legal and regulatory frameworks surrounding vaccination, including vaccine choice, vary between and sometimes within countries. This provides opportunities to model and evaluate the relationship between different policy approaches to single antigen vaccine use, vaccination acceptance, coverage, and public trust in immunization programs. Given the relatively low but non-negligible uptake of alternative vaccine schedules for non-clinical reasons in Israel, policy responses must address hesitancy with empathy and flexibility while clearly reinforcing evidence-based schedules as the norm. This is the first study in Israel to combine national vaccination data with parental, provider and policymaker perspectives, revealing how informal flexibility in service provision shapes real-world immunization behavior.

### Limitations

This study has several limitations. First, the data sources used in Israel were not specifically designed to assess the phenomenon of alternative vaccine schedules, which may have led to an underestimation of their prevalence. The study population is also unlikely to be fully representative, since families who do not attend Maternal and Child Health clinics are missing from the dataset, and these gaps are unlikely to be random. Moreover, the data of this study were derived from Maternal and Child Health Centers that cover approximately 70% of the registered child population. This means that families receiving well-child services through HMO-operated Maternal and Child Health centers were not represented in the dataset, introducing potential non-random selection that may bias both overall prevalence estimates and further limit representativeness, thereby overlooking differences in access or service delivery across providers. As a result, certain underrepresented and underserved communities may be only partially captured or absent from the dataset. Structural factors such as differential access to Maternal and Child Health center services, geographic and socioeconomic disparities, and variability in healthcare-seeking behavior may influence vaccine uptake in ways that cannot be fully assessed in this study. In addition, key covariates such as socioeconomic status and peripherality were assigned based on the clinic locality rather than the child’s place of residence, which may introduce contextual (ecological) bias. The relatively small sample size of families opting for single-antigen vaccines also limited the ability to conduct more detailed subgroup analyses. Similarly, religious and ethnic group classifications were based on administrative categories rather than self-declared identity, and include a heterogeneous “Other” category, which limits interpretability and warrants caution in cluster-based or normative interpretations. In addition, the cohort study was conducted between the years 2018–2021, and interviews were conducted in 2024, raising the possibility of temporal mismatch between quantitative patterns and qualitative accounts. Finally, the findings are specific to the Israeli healthcare system and may not be reflective of factors associated with alternative vaccine scheduling in other contexts. In addition, the categorization of (ethnic/religious group and SES level) reflects administrative and analytical classifications and may not fully capture the cultural and social heterogeneity within these populations. Although vaccination delay was operationalized using a programmatic cut-off, sensitivity analyses applying alternative thresholds and continuous measures of delay yielded consistent findings, reducing concern that the results are driven by cut-off dependence.

## 5. Conclusions

This study presents new evidence on alternative vaccination schedules in Israel, combining findings from routine data with perspectives from parents, professionals, and policymakers gathered through semi-structured interviews. Although the overall prevalence of split vaccination is low, its use is concentrated in specific socioeconomic, geographic, and religious groups. Children receiving single-antigen measles or pertussis vaccines were more likely to experience delays and incomplete series, particularly for pertussis, creating clusters of vulnerability where outbreaks may occur.

Our interview findings highlight the complexity of parental decision-making, shaped by concerns about safety, fears of “immune overload,” declining trust in the Ministry of Health after COVID-19, and misinformation circulating on social media. Some parents also relied on guidance from private or alternative practitioners. While providers sometimes agreed to split vaccines in order to maintain engagement and prevent complete refusal, these accommodations may inadvertently contribute to the normalization of non-standard schedules, create ambiguity around official recommendations, and potentially influence completion rates. Striking the right balance between these considerations, taking into account both short-term and long-term implications, remains a subject of ongoing debate across countries.

Addressing this challenge requires empathetic yet consistent communication that reinforces the safety and effectiveness of combination vaccines. Engaging trusted community figures, particularly in religious and ethnic groups, may help rebuild confidence in vaccination programs. Future mixed methods research should continue to monitor the persistence of split vaccination practices and assess their long-term implications for children’s health outcomes, as Maternal and Child Health clinics seek to understand how community norms, parental autonomy, and misinformation continue to shape vaccination decision-making.

## Figures and Tables

**Table 1:** Demographics data (general children population vs. study population).

Variable	General Population % (*N*)	Study Population % (*n*)
**Gender**		
Male	51.4% (374,568)	51.4% (256,945)
Female	48.6% (354,164)	48.6% (243,030)
**Birth Year**		
2018	25.3% (184,370)	26.2% (130,784)
2019	25.0% (182,016)	25.7% (128,568)
2020	24.3% (177,307)	24.8% (123,944)
2021	25.4% (185,040)	23.3% (116,679)
**Religion/Ethnicity**		
Jewish	60.8% (443,069)	48.5% (242,301)
Muslim	14.9% (108,581)	20.8% (103,900)
Christian	1.8% (13,117)	1.8% (8797)
Bedouin	3.0% (21,862)	3.6% (18,068)
Druze	1.3% (9473)	1.8% (8899)
Ultra-Orthodox	13.1% (95,464)	21.4% (106,972)
Other	5.0% (36,436)	1.3% (6289)
Missing	NA	0.9% (4749)
SES Status *		
1	3.8% (27,691)	7.2% (36,131)
2	17.8% (129,714)	14.8% (74,230)
3	7.6% (55,383)	23.9% (119,683)
4	4.5% (3293)	11.3% (56,587)
5	16.6% (120,969)	7.6% (37,846)
6	9.2% (67,043)	5.8% (29,024)
7	21.0% (153,033)	15.5% (82,590)
8	14.1% (102,751)	7.2% (35,964)
9	5.0% (36,436)	1.4% (7238)
10	0.3% (2186)	0.1% (255)
Missing	NA	4.1% (20,427)
**SES Subgroups**		
Low SES (1–4)	33.7% (245,583)	59.8% (286,631)
Medium SES (5–7)	46.8% (341,047)	31.2% (149,460)
High (8–10)	19.4% (141,374)	9.1% (43,457)
Peripherality Index **		
1	0.6% (4372)	1.1% (5268)
2	1.3% (9473)	0.6% (3131)
3	8.1% (59,027)	10.2% (50,904)
4	9.8% (71,415)	8.8% (43,950)
5	21.0% (153,033)	20.2% (100,752)
6	9.6% (69,958)	6.4% (31,852)
7	10.5% (76,516)	10.0% (49,851)
8	8.6% (62,671)	3.9% (19,665)
9	20.2% (147,204)	23.0% (114,774)
10	10.1% (73,602)	11.9% (59,401)
Missing		4.1% (20,427)
**Peripherality Subgroup**		
Remote Periphery (1–4)	19.8% (144,289)	21.5% (103,253)
Periphery (5–7)	41.1% (299,509)	38.0% (182,455)
Central (8–10)	38.9% (283,477)	40.4% (193,840)

* SES was determined according to the socioeconomic classification of the locality in which the Maternal and Child Health Center is located. ** The central–periphery index is an official Israeli geographic classification reflecting a locality’s distance from major metropolitan centers and its access to essential services, functioning similarly to international urban–rural classifications.

**Table 2:** Split vaccine Israel unadjusted analysis.

	Pertussis				Measles
	Dose 1	Dose 2	Dose 3	Dose 4	Dose 1
Religion/ethnic group					
General Jewish population	67.6% (341)	62.3% (426)	61.8% (311)	73.5% (89)	63.6% (1820)
Arab	5.5% (28)	6.7% (46)	6.4% (32)	4.1% (5)	6.0% (173)
Ultra-Orthodox	20.8% (105)	17.1% (117)	14.1% (71)	18.2% (22)	25.9% (742)
Others	5.9% (30)	13.9% (95)	17.7% (89)	4.1% (5)	4.5% (128)
Sig	*p* = 0.038	*p* < 0.001	*p* < 0.001	*p* > 0.05	*p* = 0.003
Socioeconomic status					
SES—Low (1–4)	46.9% (239)	45.5% (312)	43.4% (219)	36.1% (44)	46.5% (1365)
SES—Medium (5–7)	40.5% (206)	43.1% (296)	44.6% (225)	46.7% (57)	44.4% (1302)
SES—High (8–10)	12.6% (64)	11.4% (78)	11.9% (60)	17.2% (21)	9.1% (266)
Sig	*p* > 0.05	*p* > 0.05	*p* = 0.017	*p* = 0.013	*p* > 0.05
Geographical area—peripherality scale					
Far peripherality (1–4)	14.7% (75)	14.7% (101)	15.1% (76)	10.6% (13)	10.1% (297)
Peripherality (5–7)	44.6% (227)	50.1% (344)	51.4% (259)	60.0% (60)	47.1% (1380)
Center (8–10)	40.7% (207)	35.1% (241)	33.5% (169)	40.2% (49)	42.8% (1256)
Sig	*p* = 0.027	*p* > 0.05	*p* > 0.05	*p* > 0.05	*p* = 0.05
COVID-19 Time period					
Before the pandemic (2018–2019)	61.2% (335)	63.8% (387)	66.5% (278)	63.1% (115)	75.2% (2200)
During the initial pandemic phase (2020–2021)	38.8% (170)	36.2% (298)	33.5% (224)	36.9% (7)	24.8% (727)
Sig	*p* > 0.05	*p* = 0.002	*p* = 0.036	*p* > 0.05	*p* < 0.001

**Table 3:** Combination vaccine Israel unadjusted analysis.

	Pentavalent Vaccine				MMRV
	Dose 1	Dose 2	Dose 3	Dose 4	Dose 1
Religion/ethnic group					
General Jewish population	98.3%	96.8%	94.3%	87.2%	93.3%
Arab	99.5%	98.9%	97.1%	92.8%	97.7%
Ultra-Orthodox	98.2%	93.0%	86.7%	72.1%	88.5%
Others	97.3%	97.1%	95.3%	86.8%	95.3%
Sig	*p* < 0.001	*p* < 0.001	*p* < 0.001	*p* < 0.001	*p* < 0.001
Socioeconomic status					
SES—Low (1–4)	98.6%	95.7%	92.2%	83.3%	91.8%
SES—Medium (5–7)	98.1%	96.9%	95.1%	89.1%	92.5%
SES—High (8–10)	95.6%	96.7%	94.8%	89.5%	91.9%
Sig	*p* < 0.001	*p* < 0.001	*p* < 0.001	*p* < 0.001	*p* < 0.001
Geographical area—peripherality scale					
Far peripherality (1–4)	98.9%	97.0%	96.8%	88.4%	85.3%
Peripherality (5–7)	98.4%	96.4%	93.5%	85.7%	93.4%
Center (8–10)	98.6%	96.2%	92.9%	83.8%	92.7%
Sig	*p* < 0.001	*p* < 0.001	*p* < 0.001	*p* < 0.001	*p* < 0.001
COVID-19 Time period					
Before the pandemic (2018–2019)	99.0%	98.0%	96.7%	93.2%	96.4%
During the initial pandemic phase (2020–2021)	97.9%	94.7%	90.8%	81.6%	89.6%
Sig	*p* < 0.001	*p* < 0.001	*p* < 0.001	*p* < 0.001	*p* < 0.001

## Data Availability

The anonymized tabular data presented in this study are available on request from the corresponding author.

## Abbreviations

CDC	Centers for Disease Control and Prevention
DTaP+IPV+Hib	Diphtheria–Tetanus–acellular Pertussis–Inactivated Poliovirus–Haemophilus influenzae type b
HMO	Health Maintenance Organizations
MMRV	Measles-Mumps-Rubella-Varicella
OECD	Organization for Economic Co-operation and Development
UNICEF	United Nations Children’s Fund
WHO	World Health Organization
WHO SAGE	World Health Organization Strategic Advisory Group of Experts
